# Text-mining applied to autoimmune disease research: the Sjögren’s syndrome knowledge base

**DOI:** 10.1186/1471-2474-13-119

**Published:** 2012-07-03

**Authors:** Sven-Ulrik Gorr, Trevor J Wennblom, Steve Horvath, David TW Wong, Sara A Michie

**Affiliations:** 1 Department of Diagnostic and Biological Sciences, University of Minnesota School of Dentistry, Minneapolis, MN, 55455, USA; 2 Minnesota Supercomputing Institute, University of Minnesota, Minneapolis, MN, 55455, USA; 3 Department of Biostatistics, School of Public Health, University of California, Los Angeles, CA, 90095, USA; 4 School of Dentistry, University of California, Los Angeles, CA, 90095, USA; 5 Department of Pathology, Stanford University School of Medicine, Stanford, CA, 94305, USA

## Abstract

**Background:**

Sjögren’s syndrome is a tissue-specific autoimmune disease that affects exocrine tissues, especially salivary glands and lacrimal glands. Despite a large body of evidence gathered over the past 60 years, significant gaps still exist in our understanding of Sjögren’s syndrome. The goal of this study was to develop a database that collects and organizes gene and protein expression data from the existing literature for comparative analysis with future gene expression and proteomic studies of Sjögren’s syndrome.

**Description:**

To catalog the existing knowledge in the field, we used text mining to generate the Sjögren’s Syndrome Knowledge Base (SSKB) of published gene/protein data, which were extracted from PubMed using text mining of over 7,700 abstracts and listing approximately 500 potential genes/proteins. The raw data were manually evaluated to remove duplicates and false-positives and assign gene names. The data base was manually curated to 477 entries, including 377 potential functional genes, which were used for enrichment and pathway analysis using gene ontology and KEGG pathway analysis.

**Conclusions:**

The Sjögren’s syndrome knowledge base ( http://sskb.umn.edu) can form the foundation for an informed search of existing knowledge in the field as new potential therapeutic targets are identified by conventional or high throughput experimental techniques.

## Background

Sjögren’s syndrome is a tissue-specific autoimmune disease that affects exocrine tissues, especially salivary glands and lacrimal glands. It is one of the most common autoimmune disorders in the U.S., with an estimated prevalence of 2–4 million people. The autoimmune-mediated damage of the salivary and lacrimal glands in Sjögren’s syndrome leads to a decrease in the production of saliva and tears and to the development of dry mouth and dry eyes. Without the lubricating and protective functions of saliva and tears, the oral and ocular surfaces are subject to infections and discomfort, leading to a significantly reduced quality of life [1,2].

Development of Sjögren’s syndrome requires a complex interplay between a number of genetic, hormonal and environmental factors, most of which have not been defined. Genetic linkages, especially involving major histocompatibility complex (MHC) genes, have been reported for Sjögren’s syndrome but it is not clear if, or how, the associated genes are involved in the development of the disease [3]. Additional non-MHC genes have also been linked with the development of Sjögren’s syndrome.

In addition to genetic predisposition, some studies suggest that infection of a genetically-susceptible individual by a virus or other pathogen might trigger the development of an autoimmune disease [4]. The proposed mechanisms include activation of the innate immune system, release of self antigens from damaged or apoptotic tissues, and molecular mimicry that results in activation of T cells and/or B cells that react with tissue antigens [4].

Both the innate and the adaptive immune systems are involved in the pathogenesis of Sjögren’s syndrome. The type I interferon (IFN) pathway, which plays an important role in the innate immune response to viruses, is also thought to play an important role in the development of Sjögren’s syndrome and other autoimmune disorders, including SLE [5,6]. Moreover, type I IFNs can activate the adaptive immune system directly, by binding to IFN receptors on antigen presenting cells, T cells and B cells, or indirectly, by inducing the production and release of cytokines and chemokines that bind to these cells.

Autoantibodies to intracellular antigens, notably the nuclear proteins SSA/Ro and SSB/La, are found in the sera of many patients with Sjögren’s syndrome. These autoantibodies are thought to develop when intracellular antigens, some of which have undergone proteolytic cleavage that reveals new antigenic epitopes, become “visible” to the immune system in membrane blebs on the surface of apoptotic cells [7]. Alternatively, antigenic epitopes from bacteria and viruses, including Epstein-Barr virus (EBV) and coxsackie virus, may act as molecular mimics that trigger the development of antibodies that cross react with similar epitopes on target tissue autoantigens [2,8,9]. Although autoantibodies to intracellular antigens are useful in the diagnosis of Sjögren’s syndrome, it is not clear if they play a direct role in the development of salivary gland and lacrimal gland damage and hypofunction. In contrast, autoantibodies to the M3 muscarinic acetylcholine receptor (M3R) have been directly implicated in salivary gland hypofunction in the nonobese diabetic (NOD) mouse model of Sjögren’s syndrome [10]. Importantly, function-inhibiting anti-M3R autoantibodies are found in the sera of many patients with Sjögren’s syndrome [11].

Current therapy for Sjögren’s syndrome usually consists of palliative treatment that relieves the symptoms of dry eye and dry mouth, but fails to modify the underlying disease. Novel disease-modifying treatment strategies, based on recent immunological insights in Sjögren's syndrome and other autoimmune diseases, have met with mixed results [12]. For example, in recent clinical trials, treatment of Sjögren's syndrome patients with a B cell-depleting anti-CD20 monoclonal antibody (rituximab) led to significant improvement of the stimulated whole saliva flow rate and a reduction in parotid gland inflammation [13]. In contrast, TNFα inhibitors have been ineffective in the treatment of Sjögren's syndrome. Detailed studies on the immune response in Sjögren’s syndrome patients treated with one of the inhibitors (etanercept) revealed an increase in the circulating levels of TNFα [14]. These results suggest that TNFα may not play a pivotal role in the disease and that other therapeutic targets must be identified.

Despite a large body of evidence gathered over the past 60 years, significant gaps still exist in our understanding of Sjögren’s syndrome. Recent gene expression and proteomic studies have identified many genes and pathways that may play a role in the pathogenesis of Sjögren’s syndrome [15-17]. However, validation of these data will require significant additional effort. As an initial step in this validation, we have compiled the published data on Sjögren’s syndrome that is not derived from gene expression or proteomic studies. No such unifying database currently exists. Through data curation, the existing data have been uniformly formatted to allow systematic retrieval and comparisons to newly generated gene expression data. As an example of its functionality, the Sjögren's Syndrome Knowledge Base (SSKB) was analyzed for biological functions and pathways that are likely to play a role in the disease.

## Construction and content

### Data mining

To catalog the existing knowledge in the field, we used text mining to generate the Sjögren’s Syndrome Knowledge Base (SSKB) of published gene/protein data ( http://sskb.umn.edu/) [18]. The focus of this data-base is on individually identified genes and proteins. Thus, microarray experiments were not included. The raw data for SSKB was extracted from PubMed [19]) using the text mining program EBIMed ( http://www.ebi.ac.uk/Rebholz-srv/ebimed/) [20] with the search term "Sjogren's Syndrome" restricted to "MeshHeadingsList". The foundational search identified over 7,700 abstracts and approximately 500 potential genes/proteins. The SSKB is continually updated by regular automated searches of PubMed followed by manual curation.

### Curation of raw data

The identified abstracts were manually evaluated to remove duplicates and false-positives. In older publications, where gene names were not readily identifiable, names were assigned based on in depth evaluation of the protein name context and available gene data in public databases, including the National Center for Biotechnology Information’s Entrez search engine [21] and UniProt [22,23]. The SSKB includes data from human studies and animal models. For the genes identified in animal models, the human homolog was identified by automated ortholog search, using WebGestalt 2.0 [24,25]. These steps reduced the database to 477 current entries. The online database contains the fully curated data and currently contains 413 entries, which can be accessed at http://sskb.umn.edu. Updates and newly curated data are continually added.

The 477 entries were sorted to identify autoantigens and viral/bacterial antigens, resulting in 377 potential functional genes, which were used for enrichment and pathway analysis.

### Enrichment analysis

The 377 human gene entries were used for subsequent enrichment analyses in Webgestalt [24,25]. Gene enrichment in the SSKB gene set was compared to the human genome using the hypergeometric test with multiple test adjustment [26] and a significance level of P <0.01.

The Gene Ontology [27,28] was accessed with Webgestalt and analysis was restricted to processes and functions represented by two or more genes. Pathway analysis was performed with Webgestalt in the Kyoto Encyclopedia of Genes and Genomes (KEGG) [29,30]. The selection was restricted to pathways with 4 or more genes represented, resulting in identification of 72 KEGG pathways. The “salivary secretion” pathway (KO04970) was recently added to KEGG (11/9/10) and was not included in this analysis. This pathway contains 59 genes, seven of which are found in the SSKB gene set.

## Utility and discussion

We constructed a database containing proteins and genes associated with Sjögren’s syndrome in human disease or animal models, as identified by text mining of published data. The public SSKB currently contains 413 genes/proteins and can be viewed online ( http://sskb.umn.edu/). All genes have been assigned gene symbols and UniProt IDs, which allows rapid retrieval of gene-specific data from external databases. The SSKB data base can be used to determine whether a list of genes is enriched with known Sjögren’s syndrome genes and one can carry out a function enrichment analysis (hypergeometric distribution). Individual genes and the corresponding gene products, synonyms and alternate names can be searched by using a web browser search function. Autoantigens, viral antigens and bacterial antigens are separately identified under “Antigens”. The SSKB is continually maintained and updated and new genes are added as their analysis is completed.

Based on the abstracts used to retrieve the SSKB genes/proteins, 85 proteins were initially characterized as autoantigens and 15 proteins were characterized as viral (14) or bacterial (1) antigens. Not surprisingly, SSA/Ro and SSB/La were among the most frequently retrieved autoantigens. It has been proposed that viral or bacterial antigens act as autoimmune triggers by molecular mimicry of endogenous human proteins [2,8,9]. However, eight of the 14 putative viral antigens in SSKB were selected for BLAST analysis, which did not identify strong sequence similarity with human proteins (not shown).

The 377 proteins not identified as autoantigens or microbial antigens were considered candidates for functional genes that could play a role in the initiation and progression of Sjögren’s syndrome. Since the gene list contains data from humans and animals, the corresponding human genes were identified, with the assumption that genes identified in animal models of Sjögren’s syndrome may also be involved in the human disease.

### Gene ontology

The Gene Ontology database [27] was queried to identify the biological processes, cellular components and molecular functions associated with genes in the SSKB (Table 1). The 40 most highly enriched entries were identified in each category.

**Table 1:** Gene Ontology enrichment analysis

**Rank**	**BIOLOGICAL PROCESS**	**GO ID**	**Reference Genes**	**Observed Genes**	**Ratio**
1	regulation of lymphocyte proliferation	GO:0050670	81	32	39.51%
2	regulation of leukocyte proliferation	GO:0070663	82	32	39.02%
3	regulation of mononuclear cell proliferation	GO:0032944	82	32	39.02%
4	adaptive immune response based on somatic recombination of immune receptors built from immunoglobulin superfamily domains	GO:0002460	112	38	33.93%
5	adaptive immune response	GO:0002250	113	38	33.63%
6	lymphocyte proliferation	GO:0046651	112	37	33.04%
7	leukocyte proliferation	GO:0070661	114	37	32.46%
8	mononuclear cell proliferation	GO:0032943	114	37	32.46%
9	regulation of lymphocyte activation	GO:0051249	141	42	29.79%
10	regulation of cell activation	GO:0050865	168	46	27.38%
11	regulation of leukocyte activation	GO:0002694	159	43	27.04%
12	positive regulation of immune system process	GO:0002684	229	60	26.20%
13	regulation of immune response	GO:0050776	218	54	24.77%
14	immune effector process	GO:0002252	200	45	22.50%
15	regulation of immune system process	GO:0002682	362	79	21.82%
16	lymphocyte activation	GO:0046649	272	59	21.69%
17	leukocyte activation	GO:0045321	324	66	20.37%
18	inflammatory response	GO:0006954	359	71	19.78%
19	cell activation	GO:0001775	366	71	19.40%
20	immune response	GO:0006955	750	133	17.73%
21	regulation of response to stimulus	GO:0048583	441	75	17.01%
22	defense response	GO:0006952	657	100	15.22%
23	immune system process	GO:0002376	1066	162	15.20%
24	response to wounding	GO:0009611	560	85	15.18%
25	response to external stimulus	GO:0009605	904	110	12.17%
26	multi-organism process	GO:0051704	668	79	11.83%
27	regulation of programmed cell death	GO:0043067	812	92	11.33%
28	regulation of apoptosis	GO:0042981	805	91	11.30%
29	regulation of cell death	GO:0010941	815	92	11.29%
30	regulation of cell proliferation	GO:0042127	739	79	10.69%
31	apoptosis	GO:0006915	1063	102	9.60%
32	programmed cell death	GO:0012501	1071	102	9.52%
33	response to chemical stimulus	GO:0042221	1243	117	9.41%
34	cell proliferation	GO:0008283	1056	98	9.28%
35	death	GO:0016265	1171	107	9.14%
36	cell death	GO:0008219	1167	106	9.08%
37	response to stress	GO:0006950	1696	144	8.49%
38	positive regulation of biological process	GO:0048518	1865	153	8.20%
39	positive regulation of cellular process	GO:0048522	1699	130	7.65%
40	response to stimulus	GO:0050896	3471	221	6.37%
**Rank**	**CELLULAR COMPONENT**	**GO ID**	**Count**	**Observed**	**Ratio**
1	calcineurin complex	GO:0005955	5	3	60.00%
2	external side of plasma membrane	GO:0009897	131	40	30.53%
3	platelet alpha granule lumen	GO:0031093	41	12	29.27%
4	MHC class II protein complex	GO:0042613	14	4	28.57%
5	nerve terminal	GO:0043679	14	4	28.57%
6	cytoplasmic membrane-bounded vesicle lumen	GO:0060205	44	12	27.27%
7	vesicle lumen	GO:0031983	46	12	26.09%
8	integrin complex	GO:0008305	29	7	24.14%
9	platelet alpha granule	GO:0031091	52	12	23.08%
10	high-density lipoprotein particle	GO:0034364	24	5	20.83%
11	MHC protein complex	GO:0042611	38	7	18.42%
12	plasma lipoprotein particle	GO:0034358	34	6	17.65%
13	protein-lipid complex	GO:0032994	34	6	17.65%
14	cell surface	GO:0009986	305	51	16.72%
15	axon part	GO:0033267	48	7	14.58%
16	extracellular space	GO:0005615	670	84	12.54%
17	receptor complex	GO:0043235	113	13	11.50%
18	secretory granule	GO:0030141	174	19	10.92%
19	membrane raft	GO:0045121	131	14	10.69%
20	extracellular region part	GO:0044421	939	94	10.01%
21	axon	GO:0030424	148	14	9.46%
22	cell soma	GO:0043025	155	13	8.39%
23	soluble fraction	GO:0005625	297	24	8.08%
24	cytoplasmic vesicle part	GO:0044433	177	13	7.34%
25	extracellular region	GO:0005576	1984	143	7.21%
26	basolateral plasma membrane	GO:0016323	190	13	6.84%
27	lysosome	GO:0005764	206	14	6.80%
28	integral to plasma membrane	GO:0005887	1183	72	6.09%
29	intrinsic to plasma membrane	GO:0031226	1206	73	6.05%
30	cytoplasmic membrane-bounded vesicle	GO:0016023	537	32	5.96%
31	membrane-bounded vesicle	GO:0031988	555	32	5.77%
32	extracellular matrix	GO:0031012	335	19	5.67%
33	neuron projection	GO:0043005	318	18	5.66%
34	plasma membrane part	GO:0044459	1918	104	5.42%
35	cell fraction	GO:0000267	1039	55	5.29%
36	cytoplasmic vesicle	GO:0031410	628	33	5.25%
37	vesicle	GO:0031982	655	33	5.04%
38	insoluble fraction	GO:0005626	803	34	4.23%
39	plasma membrane	GO:0005886	3650	139	3.81%
40	cytosol	GO:0005829	1251	47	3.76%
**Rank**	**MOLECULAR FUNCTION**	**GO ID**	**COUNT**	**Observed**	**RATIO**
1	arginine binding	GO:0034618	3	3	100.00%
2	nitric-oxide synthase activity	GO:0004517	3	3	100.00%
3	tetrahydrobiopterin binding	GO:0034617	3	3	100.00%
4	C-X-C chemokine binding	GO:0019958	8	4	50.00%
5	beta-amyloid binding	GO:0001540	13	5	38.46%
6	tumor necrosis factor receptor binding	GO:0005164	21	8	38.10%
7	chemokine activity	GO:0008009	47	17	36.17%
8	chemokine receptor binding	GO:0042379	49	17	34.69%
9	coreceptor activity	GO:0015026	19	6	31.58%
10	tumor necrosis factor receptor superfamily binding	GO:0032813	31	9	29.03%
11	cytokine receptor binding	GO:0005126	178	46	25.84%
12	chemokine binding	GO:0019956	26	6	23.08%
13	cytokine activity	GO:0005125	196	45	22.96%
14	growth factor receptor binding	GO:0070851	67	14	20.90%
15	collagen binding	GO:0005518	35	7	20.00%
16	G-protein-coupled receptor binding	GO:0001664	107	20	18.69%
17	integrin binding	GO:0005178	58	9	15.52%
18	cysteine-type endopeptidase activity	GO:0004197	71	10	14.08%
19	growth factor activity	GO:0008083	161	19	11.80%
20	cytokine binding	GO:0019955	108	12	11.11%
21	protein heterodimerization activity	GO:0046982	189	21	11.11%
22	glycosaminoglycan binding	GO:0005539	139	14	10.07%
23	protein complex binding	GO:0032403	196	19	9.69%
24	receptor binding	GO:0005102	856	83	9.70%
25	receptor signaling protein activity	GO:0005057	159	15	9.43%
26	pattern binding	GO:0001871	153	14	9.15%
27	peptidase inhibitor activity	GO:0030414	154	14	9.09%
28	carbohydrate binding	GO:0030246	349	29	8.31%
29	endopeptidase activity	GO:0004175	370	28	7.57%
30	polysaccharide binding	GO:0030247	153	14	9.15%
31	protein dimerization activity	GO:0046983	514	36	7.00%
32	identical protein binding	GO:0042802	618	38	6.15%
33	enzyme binding	GO:0019899	505	29	5.74%
34	peptidase activity	GO:0008233	563	30	5.33%
35	peptidase activity, acting on L-amino acid peptides	GO:0070011	546	29	5.31%
36	molecular transducer activity	GO:0060089	2116	98	4.63%
37	signal transducer activity	GO:0004871	2116	98	4.63%
38	receptor activity	GO:0004872	1674	71	4.24%
39	protein binding	GO:0005515	8041	280	3.48%
40	binding	GO:0005488	12465	320	2.57%

The table ranks the gene enrichment in *biological processes*, *cellular component* and *molecular function* with corresponding GO IDs. For each GO ID, the number of *Observed Genes* identified in the SSKB was divided by the number of *Reference Genes* in the human genome to calculate the Ratio of enrichment (Ratio).

The most highly enriched *biological processes* (19 of 40; 18 of the top 20) were associated with immune function, including leukocyte proliferation, leukocyte activation, and regulation of the immune response. Other prominent biological processes were associated with apoptosis and cell death. Thus, the SSKB data set is consistent with recent microarray data [16] and reflects current models for the biological processes involved in the pathogenesis of Sjögren's syndrome [5,31,32].

The most highly enriched *cellular component* was the calcineurin complex, which plays a major role in the activation of T cells. Interestingly, in placebo-controlled clinical trials, treatment of Sjögren’s syndrome patients with eye drops that contain the calcineurin inhibitor cyclosporine, led to significant improvement in several of the signs and symptoms of dry eye [33].

Other highly enriched *cellular components* include: 1) platelet alpha granules. Although platelet activation has been reported in the salivary glands of Sjögren's syndrome patients [34], a direct search of PubMed for “platelet alpha granules” with “sjogren’s” did not retrieve any published studies. Thus, while the proteins identified were retrieved from the literature, their potential association with platelet alpha granules in Sjögren’s syndrome has not previously been noted. 2) MHC protein complexes were identified and are presumably involved in the presentation of autoantigens [16]. 3) The finding that protein-lipid complexes and lipoprotein particles are associated with Sjögren's syndrome may be consistent with changes in serum lipid levels in Sjögren's syndrome patients [35] although the prevalence of anti-phospholipid antibodies is low in Sjögren's syndrome [36]. 4) Nerve terminals and axons were also prominent cellular components, consistent with the known neurological component of Sjögren's syndrome [37].

In *molecular function*, nitric oxide synthase (NOS) activity was the most highly enriched, although only three genes (NOS1-3) were identified. Nitric oxide (NO) signaling appears to be directly affected in salivary and lacrimal glands in Sjögren’s syndrome [38]. Other highly enriched molecular functions include chemokine and cytokine activity/receptor binding (8 of the top 15) and peptidase activities.

### Pathway analysis

The SSKB gene list was submitted to KEGG [29] to identify biological pathways potentially associated with Sjögren’s syndrome. A total of 72 KEGG pathways showed highly significant enrichment (P <0.001) in this analysis (Table 2).

**Table 2:** Biological pathways associated with SSKB genes

**Rank**	**PATHWAY**	**SSKB Genes**	**ENRICHMENT**	**Raw P**	**Adjust P**
1	Allograft rejection	23	76.02	3.62E-39	6.82E-38
2	Intestinal immune network for IgA production	27	67.82	7.26E-44	2.05E-42
3	Asthma	14	58.61	4.14E-22	2.75E-21
4	Type I diabetes mellitus	20	57.09	9.13E-31	9.38E-30
5	Graft-versus-host disease	18	53.83	3.21E-27	2.79E-26
6	Autoimmune thyroid disease	22	52.13	1.29E-32	1.82E-31
7	Primary immunodeficiency	14	50.24	6.38E-21	3.79E-20
8	Hematopoietic cell lineage	33	47.1	1.39E-46	5.24E-45
9	Toll-like receptor signaling pathway	37	46.01	1.13E-51	6.38E-50
10	Apoptosis	25	35.68	5.55E-32	6.97E-31
11	NOD-like receptor signaling pathway	17	34.44	7.61E-22	4.78E-21
12	Amyotrophic lateral sclerosis (ALS)	14	33.18	5.81E-18	2.85E-17
13	Other glycan degradation	4	31.4	6.67E-06	1.24E-05
14	Cytokine-cytokine receptor interaction	66	31.05	5.91E-79	6.68E-77
15	T cell receptor signaling pathway	26	30.24	4.12E-31	4.66E-30
16	RIG-I-like receptor signaling pathway	17	30.07	9.98E-21	5.64E-20
17	Cell adhesion molecules (CAMs)	32	29.99	6.40E-38	1.03E-36
18	Bladder cancer	10	29.9	1.06E-12	3.24E-12
19	Viral myocarditis	17	29.25	1.68E-20	9.04E-20
20	Cytosolic DNA-sensing pathway	13	29.16	5.78E-16	2.42E-15
21	Pancreatic cancer	15	26.17	1.88E-17	8.50E-17
22	Small cell lung cancer	16	23.92	7.32E-18	3.45E-17
23	Glycosaminoglycan degradation	4	23.92	2.13E-05	3.65E-05
24	Natural killer cell mediated cytotoxicity	25	22.92	1.06E-26	8.56E-26
25	ErbB signaling pathway	13	22.16	2.51E-13	8.86E-13
26	Epithelial cell signaling in Helicobacter pylori infection	12	22.16	2.64E-13	9.04E-13
27	Complement and coagulation cascades	12	21.84	3.17E-13	1.05E-12
28	B cell receptor signaling pathway	13	21.77	3.38E-14	1.23E-13
29	Prion diseases	6	21.53	3.27E-07	6.84E-07
30	Antigen processing and presentation	15	21.17	5.49E-16	2.39E-15
31	Colorectal cancer	14	20.93	6.14E-15	2.48E-14
32	Adipocytokine signaling pathway	11	20.62	6.05E-12	1.80E-11
33	Chemokine signaling pathway	30	19.83	7.80E-30	7.35E-29
34	Prostate cancer	14	19.76	1.42E-14	5.53E-14
35	Glioma	10	19.32	1.10E-10	2.89E-10
36	Jak-STAT signaling pathway	23	18.64	1.67E-22	1.18E-21
37	Non-small cell lung cancer	8	18.61	1.13E-08	2.50E-08
38	Melanoma	10	17.69	2.71E-10	6.96E-10
39	Pathways in cancer	46	17.51	9.85E-43	2.23E-41
40	Fc epsilon RI signaling pathway	11	17.49	3.90E-11	1.05E-10
41	Chronic myeloid leukemia	10	16.75	4.74E-10	1.19E-09
42	GnRH signaling pathway	12	14.92	3.42E-11	9.43E-11
43	Leukocyte transendothelial migration	14	14.9	7.91E-13	2.48E-12
44	VEGF signaling pathway	9	14.87	1.04E-08	2.35E-08
45	Hypertrophic cardiomyopathy (HCM)	10	14.78	1.67E-09	4.10E-09
46	p53 signaling pathway	8	14.56	8.19E-08	1.75E-07
47	Endometrial cancer	6	14.49	3.65E-06	7.11E-06
48	Systemic lupus erythematosus	16	14.35	3.27E-14	1.23E-13
49	MAPK signaling pathway	30	14.01	3.15E-25	2.37E-24
50	Focal adhesion	22	13.75	1.21E-18	6.21E-18
51	Dilated cardiomyopathy	10	13.65	3.66E-09	8.44E-09
52	Type II diabetes mellitus	5	13.36	3.63E-05	6.12E-05
53	Neurotrophin signaling pathway	13	12.96	3.17E-11	8.96E-11
54	ECM-receptor interaction	8	11.96	3.85E-07	7.91E-07
55	Alzheimer's disease	16	11.89	6.32E-13	2.04E-12
56	Lysosome	11	11.81	2.86E-09	6.73E-09
57	Arginine and proline metabolism	5	11.63	7.15E-05	0.0001
58	Renal cell carcinoma	6	10.77	2.09E-05	3.63E-05
59	Long-term depression	6	10.77	2.09E-05	3.63E-05
60	Long-term potentiation	6	10.77	2.09E-05	3.63E-05
61	Proteasome	4	10.47	0.0006	0.0009
62	Progesterone-mediated oocyte maturation	7	10.22	6.00E-06	1.15E-05
63	TGF-beta signaling pathway	7	10.11	6.48E-06	1.22E-05
64	Regulation of actin cytoskeleton	16	9.3	2.69E-11	7.79E-11
65	Calcium signaling pathway	13	9.17	2.36E-09	5.67E-09
66	Wnt signaling pathway	11	9.15	4.17E-08	9.06E-08
67	Gap junction	6	8.37	8.67E-05	0.0001
68	Cell cycle	8	7.85	9.32E-06	1.70E-05
69	Oocyte meiosis	7	7.71	3.80E-05	6.31E-05
70	Axon guidance	7	6.82	8.33E-05	0.0001
71	Endocytosis	10	6.72	2.93E-06	5.81E-06
72	Metabolic pathways	26	2.96	1.12E-06	2.26E-06

The table lists the number of *SSKB genes* associated with individual KEGG pathways. The pathways are ranked according to their *Enrichment* relative to the number of reference genes in the human genome based on the hypergeometric test. The *raw P*-values (hypergeometric test) and the multiple test-*adjusted P*-values are listed for each pathway.

The pathway analysis revealed dominant pathways associated with immune regulation. Indeed, the eight most highly enriched pathways were associated with antigen presenting cells and activation of T cells and B cells.

Several cancer associated pathways were identified. This is partly due to the overlap between cancer pathways. These pathways typically include cytokine or growth factor stimulation of cell cycle and cell death and were not further analyzed.

Pathways associated with apoptosis, cytokine signaling and inflammation were also highly enriched. To focus on the events associated with initiation of Sjögren's syndrome, we analyzed pathways with known triggers. Several of the highly enriched pathways are triggered by bacterial toxins, viral DNA, or viral RNA. These include signaling pathways for Toll-like receptor, NOD-like receptor, RIG-I-like receptor signaling pathways and the cytosolic DNA-sensing pathway.

### Overlap with other autoimmune diseases

The KEGG pathways include several pathways for autoimmune diseases, including type I diabetes mellitus, autoimmune thyroid disease, and SLE. While about 50% of the genes associated with the first two pathways are also associated with Sjögren's syndrome, only 16 Sjögren's syndrome genes were identified in the 140-gene SLE pathway (KEGG ID: hsa05322). These findings suggest that significant differences exist in the pathogenesis of autoimmune diseases.

## Conclusions

The results of this analysis can serve as a background and comparison for the increasing number of gene expression data sets available for Sjögren’s syndrome, e.g. [15-17]. Preliminary analysis of such data sets suggest that the biological pathways identified in the SSKB are very similar to those identified in human parotid tissue but quite different from those identified in human labial salivary glands [15]. Future analyses will further define these differences and focus on the comparison of biological pathways identified in human tissues and mouse models of Sjögren’s syndrome. It is envisioned that the SSKB data can also serve as the starting point for literature reviews and literature-based validation of identified genes; functional gene enrichment studies; protein-protein interaction networks and other bioinformatics analyses; it can be used to arrive at gene sets for SNP set enrichment analysis (pathway based GWAS studies); it can be used to define a gene set for gene set enrichment analysis (GSEA); as a starting point for bioinformatics analysis protein-protein interaction networks (based on yeast 2 hybrid) can be identified among the SSKB genes.

## Availability and requirements

The Sjögren’s syndrome knowledge base is freely available at sskb.umn.edu.

## Competing interests

Dr. David Wong is scientific advisor to RNAmeTRIX Inc., a molecular diagnostics company. The authors declare no conflicts of interest.

## Authors’ contributions

SUG collected and organized data and performed data analysis and drafted the manuscript. TW designed and implemented the database and web site. DTWW contributed to data analysis, critical review of the database and editing of the manuscript. SH contributed to critical review of the database, statistical analysis and editing the manuscript. SM contributed to data analysis, critical review of the database and drafting the manuscript. All authors read and approved the final manuscript.

## Pre-publication history

The pre-publication history for this paper can be accessed here:

http://www.biomedcentral.com/1471-2474/13/119/prepub
